# Effects of an Unblanched Peanut and/or Peanut Skin Diet on Egg Quality, Egg Lipid Chemistry, and Performance of Hens Housed in a Cage-Free Environment †

**DOI:** 10.3390/ani15182673

**Published:** 2025-09-12

**Authors:** Ondulla T. Toomer, Thien C. Vu, Rebecca Wysocky, Vera Moraes, Ramon Malheiros, Quentin D. Read, Kenneth E. Anderson

**Affiliations:** 1 Food Science & Market Quality and Handling Research Unit (FSMQHRU), Agricultural Research Service, U.S. Department of Agriculture, Raleigh, NC 27695, USA; thien.vu@usda.gov; 2 Prestage Department of Poultry Science (PDPS), NC State University (NCSU), Raleigh, NC 27695, USA; becca_wysocky@ncsu.edu (R.W.); vmmoraes@ncsu.edu (V.M.); rdmalhei@ncsu.edu (R.M.); ken_anderson@ncsu.edu (K.E.A.); 3 Southeast Area, Agricultural Research Service, U.S. Department of Agriculture, Raleigh, NC 27695, USA; quentin.read@usda.gov

**Keywords:** peanuts, peanut skins, layers, poultry diets, cage-free housing environment

## Abstract

Peanuts and poultry production predominate within the southeast US. Hence, the objective of this study was to determine the dietary effects of unblanched (skin-intact) high-oleic peanuts (HOPNs) and peanut skins (PN skins) on layer performance, egg quality, and fatty acid profile. To meet these objectives, 200 hens were randomly assigned to four treatments, housed in floor pens, and fed ad libitum for 8 weeks a control, HOPN (24%), PN Skin (3%), or 2.5% oleic acid (OA) diet. All empirical data was statistically analyzed for variance at the *p* < 0.05 level. Hens fed the HOPN and PN Skin diets had heavier body weights relative to the controls at week 8 (*p* < 0.05). Layers fed the HOPN treatment had poor FCR and smaller egg weights with lower levels of polyunsaturated fatty acids relative to the controls (*p* < 0.05). Hens fed the PN Skin treatment had similar performance parameters, egg quality, and egg chemistry relative to the controls over the 8-week study. These results imply that PN skins may be an acceptable poultry feed additive without adverse effects on performance, egg quality, or egg lipid chemistry. Also, these results demonstrate the potential value-added utilization of PN skins, a waste by-product of the peanut industry, as a layer feed ingredient.

## Simple Summary

Peanut skins are an abundant processing by-product of the peanut industry, with few identified uses. We aimed to conduct a feeding trial to examine the use of peanut skins as a value-added poultry feed component in layer diets. Hens were fed for 8 weeks a conventional diet or a conventional diet supplemented with either 3% peanut skins, 2.5% monounsaturated fatty acids, or 24% unblanched peanuts. Hens fed the peanut skins and whole-peanut-containing diets had increased body weights as compared with conventionally fed birds. Hen performance (total eggs produced, feed intake), egg quality, and egg lipid chemistry were similar between the conventional and peanut-skin-supplemented treatment groups. This study supports the potential use of peanut skins within the poultry feed industry and animal food production markets.

## 1. Introduction

Previous layer feeding trials have demonstrated the effective utilization of unblanched high-oleic peanuts (20%) and oleic acid (2.6%) as alternative feed ingredients to the use of soybean meal and soy oil in layer rations [1]. However, few studies have aimed to investigate the use of peanut skins as an alternative layer feed ingredient or additive.

Annually, approximately 60% of the U.S. domestic peanut crop is utilized for peanut butter production and 15% is crushed for oil [2], with the remaining defatted peanut meal, testae (peanut skins), and hulls remaining as processing by-products. Of these by-products, peanut meal has traditionally been utilized for animal feed [3], leaving few identified uses for the remaining peanut skins and hulls. Pesti et al. (2003) demonstrated that a range of 21% to 35% peanut meal in layer diets replacing soybean meal was suitable for acceptable layer performance and egg quality with no adverse effects on animal health [4]. Nonetheless, peanut skins rich in procyanidins and proanthocyanidins are underutilized, with an excess of a million tons produced annually worldwide [5]. Moreover, peanut skins provide an available source of dietary energy (crude fat and carbohydrates), dietary protein, and antioxidants [5,6].

Previous poultry feeding trials reported that layers fed a 3% peanut skin diet (g/g) and housed in a caged environment [7] and broilers fed a 4% peanut skin diet and housed in floor pens [8] had similar performance and quality of eggs and poultry meat, respectively, compared with conventionally fed birds. Nonetheless, other studies have shown that conventional eggs produced by hens fed a conventional diet and housed in a cage-free environment had reduced egg weights and egg quality parameters (Haugh Units) as compared with conventional eggs produced by hens fed a conventional diet and housed in battery cages [9]. For this reason, we aimed to determine the effects of feeding a 3% peanut skin diet to layers housed in a cage-free housing environment (floor pens) on performance, egg quality, and egg lipid profile. New identified uses for peanut skins as an alternative poultry feed additive would add value to a sizable agricultural processing by-product while supporting sustainable animal food production. We conjecture that the inclusion of peanut skins in the diets of layers housed in a cage-free environment will not adversely impact layer performance, egg quality, or nutritional composition.

## 2. Experimental Materials and Methods

### 2.1. Experimental Design, Animal Husbandry, and Dietary Treatments

This research project and all methods and procedures were approved by the NC State University (NCSU) Institutional Animal Care and Use Committee (IACUC) (protocol # 19-761-07-A, approved 10 September 2021, expired 27 November 2022).

### 2.2. Experimental Design, Animal Husbandry, Dietary Treatments, and Hen Performance

The birds used in this study were of a single strain (Hy-Line W-36) and there were no other strains mixed into the flock. Two-hundred Hy-Line W-36 hens at approximately 40 weeks of age were randomly allocated to one of four isonitrogenous (18% crude protein) and isocaloric (3080 kcal/kg) experimental diets, with three replicates per treatment, and provided feed and water freely for 8 weeks. All experimental diets were formulated to meet and/or exceed the NRC requirements for layers. Layers were housed in floor pen environments with fresh pine shavings and fed one of the following dietary treatments: a conventional control diet, a high-oleic peanut diet (HOPN, 24% unblanched), a 3% peanut skin diet (PN Skin), or a control diet supplemented with 2.5% food-grade oleic acid (OA) oil. High-oleic peanuts were crushed using a roller mill into crumbles prior to inclusion in the finished HOPN diet. The OA diet was prepared by supplementing the control diet with 2.5% food-grade OA (Millipore Sigma, Burlington, MA, USA). Experimental diets were analyzed for lipids (total cholesterol, crude fat, fatty acid profile) by a commercial vendor (ATC Scientific, Little Rock, AK, USA).

Each treatment had three replicates with approximately 15 hens housed in each floor pen (pen dimensions 3.2 × 1.2 m) at the Animal and Poultry Teaching Education Unit, NC State University (Raleigh, NC, USA). Each replicate pen contained a nest box, a conventional hanging automatic feeder, and a conventional water bell drinker. The hens were maintained at 14:10 L:D (light 6 a.m. to 8 p.m.). All finished feed samples were analyzed by the NC Dept. of Agriculture and Consumer Services, Food and Drug Protection Division Laboratory (Raleigh, NC, USA) and determined to be free of toxins and contaminants.

Pen weights were recorded at the onset and termination (week 8) of the study. Feed weights were recorded weekly for determination of weekly feed intake. Feed conversion ratio (FCR) was calculated as kilograms of total feed consumed divided by the total number of eggs for the 8-week study. Shell eggs were collected daily and enumerated weekly. Egg quality parameters were analyzed bi-weekly (2, 4, 6, and 8 weeks) using standard methods in the Egg Quality Lab, PDPS, NCSU (Raleigh, NC, USA). The fatty acid profile, total cholesterol, and crude fat content were analyzed bi-weekly (2, 4, 6, and 8 weeks) in pooled egg samples by a commercial laboratory, ATC Scientific (Little Rock, AR, USA). Each pooled egg sample contained twelve whole eggs (4/rep) combined homogenously by mixing for 3 min in a Whirl-pak^®^ bag placed within a Smasher™ Lab Blender (Weber Scientific, Hamilton, NJ, USA). All pooled egg samples were stored for two weeks at −20 °C until analysis.

### 2.3. Egg Quality

Egg quality was assessed using a sub-sample of 120 eggs (40 eggs from each of the 3 replicates within each treatment) randomly selected from each treatment using modified methods [9,10]. Shell strength was predicted using the TA-HDplus texture analyzer (Stable Micro Systems, Surrey, UK) with a 250 kg load cell (grams of force). This texture analyzer has a trigger force of 0.02 kg and speed of 1 mm/s. Vitelline membrane strength (VMS) was determined per the manufacturer’s instructions with a 1 mm blunt probe and a 500 g load cell. The trigger force was 0.0001 kg with a 3.2 mm/s testing speed. Haugh Unit (HU) score was determined as follows: HU = 100 × Log (h − 1.7w + 7.6), with h = egg albumen height (mm) and w = weight of egg (g). HU values range from 0 to 130, and HU scores below 60 indicate un-fresh eggs [11]. Yolk color was also determined using the TSS QCD System (TSS, York, UK) yolk color scan. The yolk color scan was calibrated using the DSM Yolk Color Fan (DSM-Firmenich, Maastricht, Switzerland) that determines color density from lightest to darkest with a scale of 1 to 15 [12]. Shell color was determined using refractometry of black, blue, and red wavelengths combined to score 83.3% (white) to 0% (black).

### 2.4. Statistical Analysis

Statistical models were fit for each response variable, with fixed effects for treatment, time, and their interaction. Time in weeks since the beginning of the experiment was fit as a continuous predictor to examine linear trends in the responses. A random intercept was fit to the pen for variables that were measured at the pen level (body weight, egg weight, feed consumed, FCR, number of eggs produced). No random intercept was fit for variables that were measured at the egg level (all egg quality and fatty acid concentration variables) because eggs were pooled across pens. Because the pens were not arranged in blocks, we fit an additional fixed effect for the side of the house (left vs. right) for the variables that were measured at the pen level to account for any variation between the two sides. Variation due to the side of the house was negligible; results are presented averaged over that effect. For response variables that were measured four or more times on the same pens (egg weight, feed consumed, FCR, number of eggs produced), a first-order autoregressive (AR1) error covariance structure was fitted. Fatty acid concentrations were log-transformed before fitting the models.

All models fit were general linear models with two exceptions. The model for the number of eggs produced per week was a generalized linear mixed model with a Poisson distribution and log link function, which is appropriate for count data. The model for Roche yolk color was a cumulative logistic model with a logit link function and flexible category thresholds, which is appropriate for ordered categorical data (categories 4 and 5 were merged into a single category for this model because only one yolk in the entire dataset was scored at >4 on the Roche scale).

For all the linear models, we produced an analysis of variance table with F-tests, except for the mixed linear models, for which we produced an analysis of deviance table with chi-squared tests. We estimated marginal means for the four treatments and compared the means using Tukey post-hoc tests, adjusting 95% confidence intervals using the Sidak multiple comparison adjustment. If there was a significant interaction between treatment and time, we did a separate means comparison for each week. We also estimated the trend in the response variables over time and its 95% confidence interval, separately by treatment if there was an interaction between treatment and time.

## 3. Results

### 3.1. Chemical Composition of Feed Ingredients and Experimental Diets

Chemical analysis was conducted to determine the nutritional content of the unblanched high-oleic peanuts and peanut skins utilized as feed components in the experimental diets. Unprocessed peanut skins contain approximately 11.4% crude fiber, 20% crude protein, 23% crude fat, 2.4% ash, 5228 kcal/kg of dietary energy (Table 1), and 118 mg gallic acid equivalents/g of total phenolic compounds with potential value in animal nutrition and feed formulations. These results demonstrate that non-roasted unblanched high-oleic peanuts contain low levels of trypsin inhibitors (1600 trypsin inhibitor units/gram), with approximately 25% crude protein, 49% crude fat, and 6728 kcal/kg dietary energy (Table 1). While unprocessed soybeans provide an excellent source of crude protein (37% to 39%) and dietary energy (5200 kcal/kg), unprocessed soybeans contain high levels of trypsin inhibitors (46,733 trypsin inhibitor units/gram) and exceed the desirable trypsin inhibitor activity (TIA) threshold of 10,000 to 15,000 trypsin inhibitor units/gram for animal feed. Hence, soybeans are routinely commercially processed using solvent extraction and thermal processing treatments to extract oil and denature trypsin inhibitors to increase protein digestibility. Solvent-extracted defatted soybean meal and unprocessed unblanched high-oleic peanuts contain similar levels of crude fiber at approximately 3% (Table 1), with the conventional threshold for dietary fiber in poultry diets in a range of 2–5% [13]. Lysine, methionine, and threonine are typically the limiting amino acids found in peanuts (0.32% methionine, 0.74% threonine, 1.05% lysine) as compared with levels found in solvent-extracted defatted soybean meal (0.6–0.7% methionine, 2.0% threonine, 3.02% lysine). Methionine is the most limiting amino acid as compared to other essential amino acids in soybean meal. Thus, in this study, all experimental diets were supplemented (Table 2) with methionine (control, HOPN, PN Skin, OA), lysine (HOPN, PN Skin, OA), and threonine (HOPN diet only).

All experimental diets were formulated to be isocaloric (3080 kcal/kg), providing similar levels of metabolizable energy. Proximate analysis determined that the gross energy of the experimental diets ranged from 3100 to 3800 kcal/kg, crude fat ranged from 5.1% to 14.0% (OA, 5.1%; control, 8.4%; PN Skin, 8.7%; HOPN, 13.9%), and crude protein ranged from 19–20% to 2–3% crude fiber between the experimental diets (Table 3). The palmitic acid content in the HOPN experimental diet was 6.7% relative to 10–11% in the other experimental diets. As expected, the oleic acid content was 74.3% in the HOPN diet relative to a range of 23–28% in the other experimental diets. Omega 3 and omega 6 levels were 0.1% and 1.4%, respectively, in the HOPN experimental diet with much higher levels in the other experimental diets.

### 3.2. Layer Performance

At the onset of the study (0 weeks), there were no differences in the average body weights between the treatment groups (Table 4). However, by week 8 of the feeding trial, hens fed the 24% HOPN and 3% PN Skin experimental diets had 6.5% greater mean body weights as compared with hens of the control and OA experimental treatment groups (*p* < 0.0001). Conversely, in our previous layer feeding trial [6], hens housed in a caged environment and fed the 24% HOPN diet had significantly smaller average body weights relative to the controls and 3% PN Skin treatment groups (*p* < 0.05). Interestingly, studies have shown that, upon comparison of birds fed similar diets and housed in either a caged or floor pen housing environment, birds reared in a caged environment had restricted physical activity and significantly reduced feed intake and body weights as compared with birds raised in floor pens, which allowed birds to be more physically active, increased muscle development, increased feed consumption, and consequently yielded higher body weights [14].

Mean egg weights were lower in eggs from the HOPN group as compared with the control and PN Skin treatments, which parallels other layer feeding trials utilizing a 24% unblanched high-oleic peanut and 3% peanut skin diet [7]. However, the mean egg weights were similar between the control and PN Skin treatments at 8 weeks (*p* < 0.001). The feed conversion ratio (FCR) was adversely impacted by feeding the HOPN treatment as compared with the control-fed birds, while FCRs were similar between the control, OA, and PN Skin treatment groups (*p* < 0.01). Also, hens fed the HOPN and OA experimental diets consumed more feed as compared with control-fed hens, while the average feed intake was similar between the control and PN Skin treatment groups (*p* < 0.01) over the 8-week feeding trial (Table 4). All eggs collected from the study were classified as USDA Grade A large eggs, specified as the large egg size [15]. Furthermore, there were no significant differences in the total number of eggs produced between the treatment groups over the 8-week feeding trial (*p* > 0.05).

### 3.3. Egg USDA Grade and Quality

There were no significant differences between the experimental treatments in shell strength (SS), shell color, or albumen height egg quality parameters (Table 5). Vitelline membrane strength (VMS) was similar between the controls and all other treatment groups, while the VMS was significantly greater in HOPN eggs as compared with OA and PN Skin eggs (*p* < 0.01). The vitelline membrane plays a vital role in the structural integrity of the internal egg and separates and protects the egg yolk from the egg albumen [16]. The strength of the vitelline membrane enables the egg yolk to withstand the industrial egg breaking process, keeping the yolk separate from the egg albumen (white). In the U.S., about 3 billion pounds of liquid egg products are produced annually in egg-breaking operations in preparation of liquid egg products such as liquid egg yolks, liquid whole eggs, and liquid egg whites for use in food service [16,17]. Hence, the food service and egg-breaking industries strive for high integrity and strength of the egg vitelline membrane. The Haugh Unit (HU) was greater in HOPN eggs as compared with the controls and OA eggs, while the HU was similar between the control, OA, and PN Skin eggs (*p* < 0.05). The HU is utilized within the egg industry as the gold standard to measure egg freshness with farm-fresh eggs having a typical HU between 75–85 HUs [17]. All eggs produced in this study were very fresh with an average HU ranging between 87 and 90 HUs.

Surprisingly, the yolk color was the darkest in eggs produced by the control-fed hens as compared with the HOPN and OA treatment groups, while the egg yolk color was similar between the PN Skin and control treatments (*p* < 0.0001). In parallel to our previous studies with layers housed in battery cages and fed similar diets, the egg yolk color was similar between eggs produced by hens fed the control and PN Skin treatments, while eggs produced by hens fed the HOPN treatment had reduced egg yolk color as compared with the controls [7]. In contrast, other feeding trials with hens housed in conventional battery cages reported that hens fed a conventional corn/soy diet with 10% corn gluten meal and unblanched high-oleic peanuts produced eggs with enhanced egg yolk color as compared with conventional eggs [18]. Corn gluten meal and yellow corn are both feed ingredients rich in the yellow xanthophyll pigments that are deposited in the egg yolks, skin, and shank of poultry when fed in the diet [19].

### 3.4. Egg Beta-Carotene and Fatty Acid Profile

Egg levels of β-carotene and palmitic acid (C16:0) were similar between all treatment groups over the 8-week feeding trial (Table 6, *p* > 0.05). Eggs from hens fed the HOPN treatment had reduced levels of myristic acid (C14:0), relative to the OA (4-week) and OA and PN Skin (6-week) treatments, with similar levels between HOPN and control eggs (*p* < 0.05). Egg stearic acid (C18:0) levels were lower in the HOPN treatment group as compared with the PN Skin treatment group, while means were similar between the controls, HOPN, OA, and PN Skin treatments at week 6 only (*p* < 0.05). At week 6, eggs produced by hens fed the HOPN treatment had significantly enhanced levels of oleic acid (C18:1) compared with the controls, while levels were similar between the control, OA, and PN Skin treatments (*p* < 0.05). At week 4 and 6, eggs from hens fed the OA treatment had palmitoleic acid (C16:1) levels that were significantly higher than the other treatment groups (*p* < 0.0001), while at week 8 palmitoleic acid levels were higher in eggs produced by hens fed the OA treatments as compared with the control and HOPN treatments (*p* < 0.0001). Palmitoleic acid is an omega 7 monounsaturated fatty acid naturally occurring in high levels in macadamia plants and sea buckthorn oils [20] shown to reduce inflammation and improve blood lipid profiles with dietary supplementation in a murine model of hyperlipidemia. However, few published nutrition studies have been conducted investigating the dietary effects of palmitoleic acid supplementation with the consumption of enriched foods.

There were no significant differences in the egg gadoleic acid (C20:1) or behenic acid (C22:0) content between the treatments over the course of the study (Table 7, *p* > 0.05). Levels of linoleic acid (C18:2) in control eggs were similar to eggs from the other treatment groups, while linoleic acid levels in PN Skin eggs were significantly higher as compared with HOPN eggs (*p* < 0.01). At week 6 (*p* < 0.0001) and 8 (*p ≤* 0.01), linoleic acid levels were similar between control, OA, and PN Skin eggs, while linoleic acid levels were significantly lower in HOPN relative to the control and PN Skin treatments. At week 4, 6, and 8, linolenic acid (C18:3) levels were significantly higher in control and PN Skin eggs as compared with the other treatments, while levels were similar between the HOPN and OA treatments (*p* < 0.0001). Linoleic and linolenic acid are both essential fatty acids that have been shown to have distinct health benefits, including lower cholesterol, improved insulin sensitivity, and lower risk of heart disease and stroke [21,22,23]. Linoleic acid (an omega 6 fatty acid) is abundant in vegetable cooking oils, nuts, and seeds [22,23], while linolenic acid (an omega 3 fatty acid) is found in flaxseeds, walnuts, and chia seeds [21].

At week 2 and 4, arachidonic acid levels (C20:4) were notably higher in control eggs as compared with the other treatment groups (*p* < 0.0001). Arachidonic acid is commonly found in poultry, animal organs, meat, fish, seafood, and eggs, is an essential polyunsaturated fatty acid found embedded within the cell phospholipid membrane, and plays a critical role in cell pathways involved in inflammation, pain, and other physiological processes [24].

At week 2 and 4, brassic acid (C22:2) levels were significantly higher in control and PN Skin eggs as compared with the HOPN and OA treatments, while levels were similar between the HOPN and OA groups (*p* < 0.01). While there is a paucity of published research trials investigating the dietary effects of brassic acid, human clinical trials have demonstrated that dietary supplementation of very-long-chain monounsaturated fatty acids can enhance the gut microbiota and lower the risk of coronary artery disease [25]. Alternatively, there were no significant treatment differences in linoleic acid levels at week 2 or arachiconic and brassic acid levels at 6 and 8 weeks (Table 7).

There were no notable differences in the quantities of crude fat or lignoceric acid (C24:0) found in eggs from each of the treatment groups at any of the time points measured (Table 8, *p* > 0.05). Total omega 3 fatty acid levels were highest and similar in eggs from hens fed the control and PN Skin diets as compared to the other treatments at week 4 and 6. Alternatively, OA eggs and OA and HOPN eggs had the lowest levels of omega 3 fatty acids as compared with the control and PN Skin eggs at week 4 and week 6, respectively (*p* < 0.0001). At week 8, omega 3 fatty acid levels in control eggs were similar to levels found in OA and PN Skin eggs, while HOPN eggs had the lowest levels of omega 3 fatty acids as compared with the control and PN Skin groups (*p* < 0.0001). There were no significant differences in omega 3 or omega 6 fatty acid levels in eggs from any of the treatment groups at week 2 of the study.

At week 4 (*p* < 0.01) and week 6 (*p* < 0.0001), omega 6 fatty acid levels in eggs from the controls were similar to levels found in the OA and PN Skin treatment eggs, while HOPN eggs had lower levels of omega 6 fatty acids as compared with control and PN Skin eggs. Similarly, at week 8, omega 6 fatty acid levels in control eggs were similar to levels found in the OA and PN Skin treatment eggs, while HOPN eggs had lower levels of omega 6 fatty acids as compared with the PN Skin treatment eggs only (*p* < 0.05).

## 4. Discussion

Traditionally, soybean meal and maize have been utilized in poultry rations globally [26,27,28,29]. However, these poultry feed ingredients are costly and often inaccessible to developing countries and small producers. Consequently, numerous poultry feeding trials are conducted annually to investigate the use of affordable and effective alternative poultry feed ingredients for the production of eggs and poultry meat. Moreover, U.S. poultry producers have shifted over the last decade from “caged” to “cage-free” housing environments due to increasing concerns related to animal welfare and consumer demands [30,31,32,33]. Thus, there is a need to investigate the effect of conventional and new feeding regimens on poultry performance within alternative housing environments.

In this 8-week feeding trial, we investigated the dietary and housing effects of feeding a 3% unprocessed peanut skin diet to layers housed in a cage-free housing environment on performance, egg quality, and egg lipid profile. In summary, this study illustrates that feeding a 3% PN Skin diet to hens housed in a cage-free environment does not adversely impact layer performance, egg quality, egg beta-carotene, or lipid content. In contrast, this study does illustrate that feeding hens housed in a cage-free environment a 24% HOPN diet does adversely impact layer performance, while egg quality parameters (with the exception of yolk color) were not adversely affected. Interestingly, eggs produced from hens fed the 24% HOPN diet had similar levels of beta-carotene and lipids (with the exception of oleic acid at week 6) as compared to eggs produced from hens fed the control diet. Eggs produced from hens fed the 24% HOPN diet in general had reduced levels of omega 3 and omega 6 fatty acids as compared with conventional control eggs, which parallels previous findings [1].

While this study implies that peanut skins are a suitable feed ingredient for layer production and egg quality within a cage-free production environment, utilization of unblanched high-oleic peanuts in the diets of layers within this environment may not be suitable for layer performance. These effects might have been attributable to the slightly higher levels of crude fiber in the HOPN diet relative to the other treatment groups, which may have resulted in reduced nutrient digestibility and uptake.

## 5. Conclusions

In conclusion, this study suggests that peanut skins could potentially serve as an alternative layer feed additive and may serve to identify new uses for a considerable processing by-product of the peanut industry beyond the nation’s landfills. While this study reports positive implications for the use of peanut skins as a potential poultry feed additive, we aim to conduct future floor-pen layer feeding trials with a larger sample size and treatment replicates to better ascertain the effects on layer performance, egg quality, and egg lipid composition. We believe that identifying value-added uses for peanut skins as a poultry feed additive will also aim to promote agricultural sustainability and regional production within the U.S. southeast, a region in which peanut and poultry production predominate.

## Figures and Tables

**Table 1:** Composition of unblanched high-oleic peanuts and peanut skins ^1^.

Component	Unblanched High-Oleic Peanuts	Peanut Skins
% Crude Protein *	25.14	20.23
% Crude Fat	49.20	23.19
% Ash	2.13	2.35
% Starch	6.00	0
% Crude Fiber	2.82	11.41
Moisture	6.42	9.89
Gross Energy (kcal/kg)	6728	5228
% Histidine	0.67	0.61
% Isoleucine	0.99	0.72
% Leucine	1.81	1.22
% Lysine	1.05	1.22
% Methionine	0.32	0.24
% Phenylalanine	1.45	0.89
% Threonine	0.74	0.60
% Tryptophan	0.23	0.02
% Arginine	3.21	1.81
% Valine	1.21	0.88
% Aspartic Acid	3.09	2.00
% Threonine	0.74	0.60
% Serine	1.22	1.19
% Glutamic Acid	4.97	2.63
% Proline	1.13	0.82
Trypsin Inhibitor (TIU/g)	1600	-

^1^ Nutritional chemistry of unprocessed unblanched high-oleic peanuts and peanut skins. %W/W = grams per 100 g of sample. * Crude protein = %N × 6.25.

**Table 2:** Composition of formulated experimental layer diets.

	Treatments ^1^			
	Control	HOPN	PN Skin	OA
Ingredients			% (by Weight)	
Soybean Meal	20.4	0	12.0	10.0
Yellow Corn	47.5	36.9	56.9	57.0
High-Oleic Peanut ^2^	0	24.0	0	0.0
Soybean Oil	7.8	0	4.4	0.0
Wheat Bran	6.0	20.0	5.0	8.7
Soy Protein Isolate	5.0	5.5	7.5	7.8
Peanut Skin	0	0	3.0	0
Oleic Acid Oil	0.0	0.0	0.0	2.5
Calcium Carbonate	10.8	10.8	9.1	11.3
Dicalcium Phosphorus	1.5	1.2	1.6	1.5
Sodium Chloride	0.3	0.3	0.3	0.3
L-Lysine	0	0.5	0.1	0.2
DL-Methionine	0.2	0.3	0.3	0.2
L-Threonine	0	0.1	0	0
Choline Chloride	0.2	0.2	0.2	0.2
Santoquin^® 3^	0.1	0.1	0.1	0.1
Mineral Premix ^4^	0.2	0.2	0.2	0.1
Vitamin Premix ^5^	0.1	0.1	0.1	0.1
Selenium Premix ^6^	0.1	0.1	0.1	0.1
Moisture	9.1	9.1	9.3	9.2
Metabolizable Energy ^7^	3080	3080	3080	3080

^1^ Four experimental diets (formulated 18% CP) were fed to 200 hens (Hy-Line W-36) for 8 weeks. Treatments: conventional control diet (corn/soy), HOPN = 24% unblanched high-oleic peanut diet, PN Skin = 3.0% peanut skin diet, OA = 2.5% oleic-acid-oil-supplemented control diet. ^2^ High-oleic peanuts = unblanched raw whole high-oleic peanut crumbles. ^3^ Santoquin^®^ = Feed antioxidant and preservative to prevent fat oxidation in stored feed (Novus International, St. Charles, MO, USA). ^4^ Mineral premix manufactured by NCSU Feed Mill, supplied the following per kg of diet: manganese, 120 mg; zinc, 120 mg; iron, 80 mg; copper, 10 mg; iodine, 2.5 mg; and cobalt. ^5^ Vitamin premix manufactured by NCSU Feed Mill supplied the following per kg of diet: 13,200 IU, vitamin A, 4000 IU vitamin D3, 33 IU vitamin E, 0.02 mg vitamin B12, 0.13 mg biotin, 2 mg menadione (K3), 2 mg thiamine, 6.6 mg riboflavin, 11 mg d-pantothenic acid, 4 mg vitamin B6, 55 mg niacin, and 1.1 mg folic acid. ^6^ Selenium premix manufactured by NCSU Feed Mill = 1 mg selenium premix provided 0.2 mg Se (as Na_2_SeO_3_) per kg of diet. ^7^ Metabolizable energy = kcal/kg feed.

**Table 3:** Chemical analysis of experimental layer diets.

	Treatments ^1^			
	Control	HOPN	PN Skin	OA
Component	% (by Weight)			
Crude Fat ^2^	8.4	13.9	8.7	5.1
Crude Protein	19.4	18.5	20.2	19.0
Fiber	2.3	3.2	1.9	2.4
* Palmitic	10.8	6.7	10.2	10.8
* Stearic	3.8	3.2	3.6	2.7
* Oleic	22.6	74.3	27.8	25.9
* Elaidic	1.3	0.7	1.2	1.0
* Linoleic	52.5	7.1	48.4	45.8
* Omega 3	6.618	0.1	58.5	3.2
* Omega 6	53.2	1.4	49.4	47.8
Gross Energy ^3^	3506	3757	3308	3085

^1^ Experimental layer diets underwent proximate and fatty acid analysis by a commercial vendor (ATC Scientific, Little Rock, AR, USA). Treatments: conventional control diet (corn/soy), HOPN = 24% unblanched high-oleic peanut diet, PN Skin = 3.0% peanut skin diet, OA = 2.5% oleic-acid-oil-supplemented control diet. ^2^ Crude fat content % = g crude fat/g total sample weight × 100, * % fatty acid content = g of fatty acid/g total lipids × 100. Each value represents the average of triplicate samples. ^3^ Gross energy = kcal/kg feed.

**Table 4:** Performance parameters of hens fed a peanut skin diet ^1^.

Treatments	0 wk BW (kg)	8 wk BW (kg)	FCR ^2^ (kg Feed/Dozen Eggs)	Total Feed Consumed (kg)	Total # Eggs Produced	Egg Weight ^3^ (g)
Control	1.59	1.65 ^b^	1.35 ^b^	7.29 ^b^	529.32	61.2 ^a^
HOPN	1.58	1.76 ^a^	1.62 ^a^	9.11 ^a^	544.56	59.1 ^b^
OA	1.58	1.65 ^b^	1.51 ^ab^	8.87 ^a^	565.60	60.1 ^ab^
PN Skin	1.61	1.77 ^a^	1.41 ^ab^	8.51 ^ab^	586.25	60.6 ^a^
SEM	0.02	0.02	0.008	0.02	26.75	0.37
*p*-value *	0.98	<0.0001	0.004	0.005	0.51	0.0005

Two-hundred hens (Hy-Line W-36) were assigned to one of four isonitrogenous (18% crude protein) and isocaloric (3080 kcal/kg) diets (five replicates per treatment) and fed for 8 weeks ad libitum. Body weights (BWs) were collected at week 1 and week 8 of the study. Each value represents the experimental average for each treatment. ^1^ Treatments: conventional control (corn/soy), HOPN = 24% unblanched high-oleic peanut diet, PN Skin = 3.0% peanut skin diet, OA = 2.5% oleic-acid-oil-supplemented control diet. ^2^ Feed conversion ratio (FCR) = 8-week kg total feed intake/total number of eggs produced over 8 weeks for each treatment group. ^3^ 8-week average egg weight per treatment group. ^a,b^ Means within the same column lacking a common superscript differ significantly (*p* < 0.05). # Means word number instead of symbol. * *p*-value = differences determined by ANOVA, *p* < 0.05.

**Table 5:** Quality of eggs produced by hens fed a peanut skin diet ^1^.

Treatments	SS (g Force)	VMS (g Force)	HU	Shell Color (%)	Albumen Height (mm)	Yolk Color
Control	3930	1.80 ^ab^	87.1 ^b^	81.5	7.74	3.06 ^a^
HOPN	4090	1.89 ^a^	89.9 ^a^	81.6	8.14	1.72 ^b^
OA	3920	1.70 ^b^	86.8 ^b^	81.3	7.66	2.72 ^b^
PN Skin	4100	1.69 ^b^	88.2 ^ab^	80.9	7.92	3.00 ^ab^
SEM	76	0.045	0.75	0.30	0.13	0.14
*p*-value *	0.17	0.0051	0.016	0.34	0.071	<0.0001

Two-hundred Hy-Line W-36 hens (40 weeks of age) were assigned to one of four isonitrogenous (18% crude protein) and isocaloric (3080 kcal/kg) diets (five replicates per treatment) and fed for 8 weeks ad libitum. Egg quality analysis was conducted at weeks 0, 2, 4, 6, and 8 using a 120 sub-sample of eggs randomly selected from each treatment using a Technical Services and Supplies QCD system, with calibration with the DSM color fan for yolk color. All data represent the 8-week average of each egg quality parameter: SS (shell strength); VMS (vitelline membrane strength); HU (Haugh Unit); Albumen Height; Yolk Color (DSM color index 1 light to 15 dark color intensity). ^1^ Treatments: control = conventional soybean meal and corn mash diet, HOPN = 24% unblanched high-oleic peanut crumble and corn mash diet, PN Skin = control diet supplemented with 3.0% ground peanut skins, OA = control diet supplemented with 2.5% food-grade oleic fatty acid oil. ^a,b^ Means within the same column lacking a common superscript differ significantly (*p* < 0.05). * *p*-value = differences determined by ANOVA, *p* < 0.05.

**Table 6:** Beta-carotene and fatty acid profile of eggs produced by hens fed a peanut skin diet ^1^.

Week	Treatment	β-Carotene	% Myristic Acid (C14:0)	% Palmitic Acid (C16:0)	% Palmitoleic Acid (C16:1)	% Stearic Acid (C18:0)	% Oleic Acid (C18:1)
2	Control	4.4 ± 1.1	0.01 ± 0.002	1.4 ± 0.17	0.08 ± 0.01	0.47 ± 0.06	2.5 ± 0.40
	HOPN	4.6 ± 1.2	0.01 ± 0.002	1.3 ± 0.16	0.08 ± 0.01	0.40 ± 0.05	2.9 ± 0.46
	OA	3.8 ± 1.0	0.01 ± 0.003	1.2 ± 0.15	0.12 ± 0.02	0.36 ± 0.05	2.1 ± 0.32
	PN Skin	4.2 ± 1.1	0.01 ± 0.003	1.3 ± 0.16	0.09 ± 0.01	0.49 ± 0.07	1.9 ± 0.30
	* *p*-value	0.967	0.286	0.928	0.044	0.306	0.238
4	Control	3.6 ± 0.6	0.011 ± 0.001 ^ab^	1.4 ± 0.12	0.08 ± 0.01 ^b^	0.51 ± 0.05	2.5 ± 0.25
	HOPN	4.1 ± 0.7	0.010 ± 0.001 ^b^	1.3 ± 0.11	0.07 ± 0.01 ^b^	0.42 ± 0.04	3.3 ± 0.33
	OA	3.7 ± 0.6	0.017 ± 0.002 ^a^	1.4 ± 0.11	0.14 ± 0.01 ^a^	0.43 ± 0.04	2.5 ± 0.25
	PN Skin	4.1 ± 0.7	0.016 ± 0.002 ^ab^	1.5 ± 0.12	0.10 ± 0.01 ^b^	0.56 ± 0.05	2.2 ± 0.23
	* *p*-value	0.925	0.025	0.649	<0.00001	0.061	0.062
6	Control	3.0 ± 0.5	0.014 ± 0.002 ^ab^	1.5 ± 0.12	0.08 ± 0.01 ^bc^	0.55 ± 0.05 ^ab^	2.4 ± 0.24 ^b^
	HOPN	3.6 ± 0.6	0.011 ± 0.001 ^b^	1.4 ± 0.11	0.07 ± 0.01 ^c^	0.45 ± 0.04 ^b^	3.6 ± 0.37 ^a^
	OA	3.5 ± 0.6	0.019 ± 0.002 ^a^	1.7 ± 0.13	0.16 ± 0.01 ^a^	0.51 ± 0.05 ^ab^	2.9 ± 0.30 ^ab^
	PN Skin	4.0 ± 0.7	0.018 ± 0.002 ^a^	1.7 ± 0.14	0.11 ± 0.01 ^b^	0.65 ± 0.06 ^a^	2.5 ± 0.26 ^ab^
	* *p*-value	0.673	0.010	0.152	<0.00001	0.035	0.026
8	Control	2.5 ± 0.7	0.02 ± 0.003	1.6 ± 0.19	0.09 ± 0.01 ^bc^	0.60 ± 0.08	2.3 ± 0.36
	HOPN	3.3 ± 0.8	0.01 ± 0.002	1.4 ± 0.17	0.07 ± 0.01 ^c^	0.48 ± 0.06	4.0 ± 0.63
	OA	3.3 ± 0.8	0.02 ± 0.004	1.9 ± 0.23	0.18 ± 0.02 ^a^	0.60 ± 0.08	3.5 ± 0.54
	PN Skin	3.9 ± 1.0	0.02 ± 0.004	2.0 ± 0.24	0.13 ± 0.02 ^ab^	0.74 ± 0.10	2.9 ± 0.45
	* *p*-value	0.651	0.099	0.157	<0.00001	0.151	0.078

Each value represents the mean ± the standard error. Two-hundred Hy-Line W-36 hens (40 weeks of age) were assigned to one of four isonitrogenous (18% crude protein) and isocaloric (3080 kcal/kg) diets (five replicates per treatment) and fed for 8 weeks ad libitum. ^1^ Treatments: control = conventional soybean meal and corn mash diet, HOPN = 24% unblanched high-oleic peanut crumble and corn mash diet, PN Skin = control diet supplemented with 3.0% ground peanut skins, OA = control diet supplemented with 2.5% food-grade oleic fatty acid oil. The fatty acid profile and beta-carotene levels were analyzed bi-weekly (2, 4, 6, and 8 weeks) using pooled egg samples for each treatment. Analysis was conducted by a commercial laboratory, ATC Scientific (Little Rock, AR, USA). Each pool sample contained 12 homogenously combined whole eggs (4 eggs from each replicate). ^a,b,c^ Means within the same column lacking a common superscript differ significantly (*p* < 0.05). * *p*-value = differences determined by ANOVA, *p* < 0.05.

**Table 7:** Fatty acid profile of eggs produced by hens fed a peanut skin diet ^1^.

Week	Treatment	% Linoleic Acid (C18:2)	% Linolenic Acid (C18:3)	% Gadoleic Acid (C20:1)	% Arachidonic Acid (C20:4)	% Behenic Acid (C22:0)	% Brassic Acid (C22:2)
2	Control	0.99 ± 0.24	0.03 ± 0.02	0.02 ± 0.005	0.15 ± 0.03 ^a^	0.07 ± 0.02	0.07 ± 0.02 ^a^
	HOPN	0.66 ± 0.16	0.02 ± 0.01	0.02 ± 0.006	0.06 ± 0.01 ^b^	0.05 ± 0.01	0.02 ± 0.01 ^b^
	OA	0.61 ± 0.15	0.01 ± 0.01	0.01 ± 0.004	0.06 ± 0.01 ^b^	0.03 ± 0.01	0.02 ± 0.004 ^b^
	PN Skin	1.21 ± 0.29	0.06 ± 0.03	0.01 ± 0.003	0.05 ± 0.01 ^b^	0.09 ± 0.03	0.04 ± 0.01 ^ab^
	* *p*-value	0.146	0.045	0.484	<0.00001	0.101	0.001
4	Control	1.15 ± 0.18 ^ab^	0.05 ± 0.01 ^a^	0.02 ± 0.003	0.15 ± 0.02 ^a^	0.03 ± 0.005	0.03 ± 0.005 ^a^
	HOPN	0.64 ± 0.10 ^b^	0.01 ± 0.004 ^b^	0.02 ± 0.005	0.09 ± 0.01 ^b^	0.02 ± 0.004	0.01 ± 0.002 ^b^
	OA	0.72 ± 0.11 ^b^	0.01 ± 0.004 ^b^	0.02 ± 0.003	0.08 ± 0.01 ^b^	0.02 ± 0.003	0.01 ± 0.002 ^b^
	PN Skin	1.37 ± 0.21 ^a^	0.07 ± 0.02 ^a^	0.01 ± 0.003	0.08 ± 0.01 ^b^	0.03 ± 0.006	0.02 ± 0.003 ^ab^
	* *p*-value	0.002	<0.00001	0.174	<0.00001	0.112	0.001
6	Control	1.32 ± 0.20 ^ab^	0.06 ± 0.02 ^a^	0.02 ± 0.003	0.15 ± 0.02	0.01 ± 0.002	0.01 ± 0.002
	HOPN	0.61 ± 0.10 ^c^	0.01 ± 0.003 ^b^	0.03 ± 0.007	0.12 ± 0.01	0.01 ± 0.002	0.01 ± 0.001
	OA	0.85 ± 0.13 ^bc^	0.02 ± 0.01 ^b^	0.02 ± 0.004	0.11 ± 0.01	0.01 ± 0.002	0.01 ± 0.001
	PN Skin	1.55 ± 0.24 ^a^	0.08 ± 0.02 ^a^	0.02 ± 0.003	0.12 ± 0.01	0.01 ± 0.002	0.01 ± 0.001
	* *p*-value	<0.00001	<0.00001	0.072	0.434	0.742	0.356
8	Control	1.51 ± 0.36 ^a^	0.08 ± 0.03 ^a^	0.01 ± 0.004	0.14 ± 0.02	0.004 ± 0.001	0.004 ± 0.001
	HOPN	0.59 ± 0.14 ^b^	0.01 ± 0.003 ^b^	0.04 ± 0.013	0.17 ± 0.03	0.004 ± 0.001	0.005 ± 0.001
	OA	1.00 ± 0.24 ^ab^	0.02 ± 0.001 ^b^	0.03 ± 0.008	0.16 ± 0.03	0.004 ± 0.001	0.004 ± 0.001
	PN Skin	1.75 ± 0.41 ^a^	0.09 ± 0.004 ^a^	0.02 ± 0.006	0.19 ± 0.03	0.004 ± 0.001	0.004 ± 0.001
	* *p*-value	0.010	<0.00001	0.158	0.707	0.996	0.885

Each value represents the mean ± the standard error. Two-hundred Hy-Line W-36 hens (40 weeks of age) were assigned to one of four isonitrogenous (18% crude protein) and isocaloric (3080 kcal/kg) diets (five replicates per treatment) and fed for 8 weeks ad libitum. ^1^ Treatments: control = conventional soybean meal and corn mash diet, HOPN = 24% unblanched high-oleic peanut crumble and corn mash diet, PN Skin = control diet supplemented with 3.0% ground peanut skins, OA = control diet supplemented with 2.5% food-grade oleic fatty acid oil. The fatty acid profile was analyzed bi-weekly (2, 4, 6, and 8 weeks) using pooled egg samples for each treatment. Analysis was conducted by a commercial laboratory, ATC Scientific (Little Rock, AR, USA). Each pool sample contained 12 homogenously combined whole eggs (4 eggs from each replicate). ^a,b,c^ Means within the same column lacking a common superscript differ significantly (*p* < 0.05). * *p*-value = differences determined by ANOVA, *p* < 0.05.

**Table 8:** Crude fat and fatty acid profile of eggs produced by hens fed a peanut skin diet ^1^.

Week	Treatment	% Lignoceric Acid (C24:0)	% Crude Fat	% Total Omega 3 Fatty Acids	% Total Omega 6 Fatty Acids
2	Control	0.004 ± 0.001	6.3 ± 0.73	0.05 ± 0.02	1.24 ± 0.27
	HOPN	0.004 ± 0.011	6.0 ± 0.70	0.04 ± 0.01	0.76 ± 0.16
	OA	0.004 ± 0.011	4.7 ± 0.54	0.02 ± 0.01	0.69 ± 0.15
	PN Skin	0.004 ± 0.011	5.4 ± 0.63	0.08 ± 0.03	1.31 ± 0.28
	* *p*-value	0.959	0.286	0.093	0.085
4	Control	0.008 ± 0.011	6.5 ± 0.50	0.06 ± 0.01 ^ab^	1.39 ± 0.20 ^ab^
	HOPN	0.008 ± 0.011	6.5 ± 0.49	0.03 ± 0.01 ^bc^	0.77 ± 0.11 ^c^
	OA	0.008 ± 0.011	5.6 ± 0.42	0.02 ± 0.01 ^c^	0.83 ± 0.12 ^bc^
	PN Skin	0.009 ± 0.011	6.3 ± 0.48	0.08 ± 0.02 ^a^	1.50 ± 0.21 ^a^
	* *p*-value	0.959	0.428	<0.00001	0.001
6	Control	0.018 ± 0.002	6.8 ± 0.52	0.07 ± 0.02 ^a^	1.55 ± 0.22 ^ab^
	HOPN	0.018 ± 0.002	7.0 ± 0.53	0.02 ± 0.004 ^b^	0.77 ± 0.11 ^c^
	OA	0.017 ± 0.002	6.7 ± 0.51	0.03 ± 0.01 ^b^	0.99 ± 0.14 ^bc^
	PN Skin	0.019 ± 0.002	7.3 ± 0.56	0.09 ± 0.02 ^a^	1.72 ± 0.24 ^a^
	* *p*-value	0.868	0.837	<0.00001	<0.00001
8	Control	0.041 ± 0.008	7.1 ± 0.82	0.08 ± 0.03 ^a^	1.73 ± 0.37 ^ab^
	HOPN	0.041 ± 0.008	7.6 ± 0.88	0.01 ± 0.004 ^b^	0.78 ± 0.17 ^b^
	OA	0.034 ± 0.006	8.0 ± 0.93	0.03 ± 0.01 ^ab^	1.20 ± 0.26 ^ab^
	PN Skin	0.043 ± 0.008	8.5 ± 0.99	0.09 ± 0.03 ^a^	1.98 ± 0.43 ^a^
	* *p*-value	0.839	0.716	<0.00001	0.017

Each value represents the mean ± the standard error. Two-hundred Hy-Line W-36 hens (40 weeks of age) were assigned to one of four isonitrogenous (18% crude protein) and isocaloric (3080 kcal/kg) diets (five replicates per treatment) and fed for 8 weeks ad libitum. ^1^ Treatments: control = conventional soybean meal and corn mash diet, HOPN = 24% unblanched high-oleic peanut crumble and corn mash diet, PN Skin = control diet supplemented with 3.0% ground peanut skins, OA = control diet supplemented with 2.5% food-grade oleic fatty acid oil. The fatty acid profile and crude fat were analyzed bi-weekly (2, 4, 6, and 8 weeks) using pooled egg samples for each treatment. Analysis was conducted by a commercial laboratory, ATC Scientific (Little Rock, AR, USA). Each pool sample contained 12 homogenously combined whole eggs (4 eggs from each replicate). ^a,b,c^ Means within the same column lacking a common superscript differ significantly (*p* < 0.05). * *p*-value = differences determined by ANOVA, *p* < 0.05.

## Data Availability

All data are available upon request.

## Abbreviations

The following abbreviations are used in this manuscript:

HOPN	Unblanched high-oleic peanut
PN Skin	Peanut skin
OA	Oleic Acid
NRC	National Research Council
FCR	Feed conversion ratio
TIA	Trypsin Inhibitor Activity
HU	Haugh Unit
